# Quantum conductance-temperature phase diagram of granular superconductor K_*x*_Fe_2−*y*_Se_2_

**DOI:** 10.1038/s41598-018-25052-0

**Published:** 2018-05-04

**Authors:** C. C. Soares, M. ElMassalami, Y. Yanagisawa, M. Tanaka, H. Takeya, Y. Takano

**Affiliations:** 1 Instituto de Fisica, Universidade Federal do Rio de Janeiro, Caixa Postal 68528, 21945-970 Rio de Janeiro, Brazil; 2 MANA, National Institute for Materials Science, 1-2-1 Sengen, Tsukuba, Ibaraki 305-0047 Japan; 3 Graduate School of Engineering, Kyushu Institute of Technology, 1-1 Sensui-cho, Tobata, Kitakyushu 804-8550 Japan

## Abstract

It is now well established that the microstructure of Fe-based chalcogenide K_*x*_Fe_2−*y*_Se_2_ consists of, at least, a minor (~15 percent), nano-sized, superconducting K_*s*_Fe_2_Se_2_ phase and a major (~85 percent) insulating antiferromagnetic K_2_Fe_4_Se_5_ matrix. Other intercalated *A*_1−*x*_Fe_2−*y*_Se_2_ (*A* = Li, Na, Ba, Sr, Ca, Yb, Eu, ammonia, amide, pyridine, ethylenediamine etc.) manifest a similar microstructure. On subjecting each of these systems to a varying control parameter (e.g. heat treatment, concentration *x*,*y*, or pressure *p*), one obtains an exotic normal-state and superconducting phase diagram. With the objective of rationalizing the properties of such a diagram, we envisage a system consisting of nanosized superconducting granules which are embedded within an insulating continuum. Then, based on the standard granular superconductor model, an induced variation in size, distribution, separation and Fe-content of the superconducting granules can be expressed in terms of model parameters (e.g. tunneling conductance, *g*, Coulomb charging energy, *E*_*c*_, superconducting gap of single granule, Δ, and Josephson energy *J* = *π*Δ*g*/2). We show, with illustration from experiments, that this granular scenario explains satisfactorily the evolution of normal-state and superconducting properties (best visualized on a ${\boldsymbol{g}}{\boldsymbol{-}}\frac{{{\boldsymbol{E}}}_{{\boldsymbol{c}}}}{{\boldsymbol{\Delta }}}{\boldsymbol{-}}{\boldsymbol{T}}$ phase diagram) of *A*_*x*_Fe_2−*y*_Se_2_ when any of *x*, *y*, *p*, or heat treatment is varied.

## Introduction

Ternary Fe-based chalcogenides *A*_1−*x*_Fe_2−*y*_Se_2_ superconductors (*A* = Li, Na, Ba, Sr, Ca, Yb, Eu, K, ammonia, amide, pyridine, ethylenediamine etc.) exhibit layered tetragonal structure which results from intercalating *A* atoms into the layered FeSe superconductor^1–9^. These chalcogenides were reported to exhibit remarkable electronic states such as unconventional superconductivity, Fermi-liquid state^10^, quantum criticality^10^, orbital selective Mott phase^11–15^ and percolative conductivity^16–18^. As an illustration, consider the archetypal K_*x*_Fe_2−*y*_Se_2_ superconductor^1–3^ (the main interest of this work): Its resistivity manifests a high-temperature semiconducting-like character; on decreasing the temperature, this is followed by a coherence peak at *T*_*mt*_ ≈ 200 K with a crossover into a metallic and, afterwards, a Fermi-liquid state; on further cooling, the latter is transformed into a superconducting state at *T*_*c*_ ≈ 30–48 K. The *T*_*mt*_ event, *apparently* not accompanied by any structural or magnetic transformation^10,19^, is monotonically increased with pressure (<9 GPa)^10,20^. Actually, pressure was reported to induce a strong and monotonic suppression in the high-temperature semiconducting-like behavior, in the Fermi-liquid character as well as in *T*_*c*_: A hint, as we shall verify below, that all these electronic states are strongly correlated^10,20^.

There are two additional remarkable properties of K_*x*_Fe_2−*y*_Se_2_: a nonstoichiometry in both K and Fe and a segregation into at least two phases^17,21–24^, namely (i) a minor K_*s*_Fe_2_Se_2_ which is a *nano-sized* and Fe-rich superconductor (denoted as K_*s*_Fe_2_Se_2_ following the convention of refs^16,17,22^ and (ii) a major K_2_Fe_4_Se_5_ which is a vacancy-ordered antiferromagnetic semiconductor. It is worth mentioning that such a phase segregation had been confirmed by various studies such as diffraction^17,23,25^, X-ray spectroscopy^26,27^, Mössbauer spectroscopy^28^, and electron microscopy imaging^21,29–32^.

**Figure 1:**
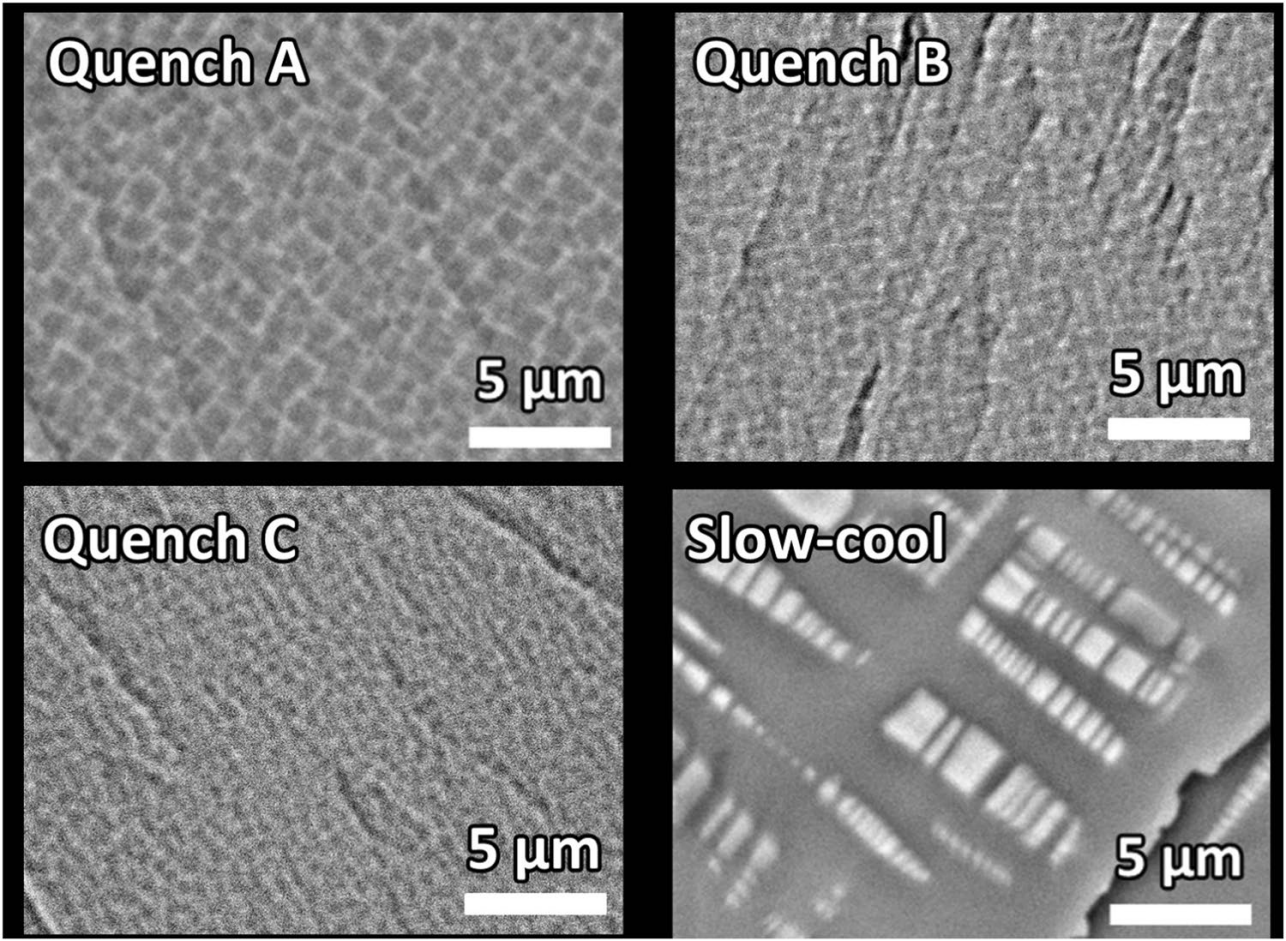
Back-scattered electron images of SEM measurements on freshly cleaved surface of each of the four samples^22,29,40^. Granular character is manifested as nano-sized stripe-like bright area (metallic K_*s*_Fe_2_Se_2_ granules) that are embedded within a dark background (insulating K_2_Fe_4_Se_5_ continuum). These images are in good agreement with the ones reported in refs^21,30^ (and references therein). The area of the uniformly distributed bright mesh-like texture in quenched samples becomes finer along *A* → *B* → *C*. Based on panels of Fig. 2(b–e), bright area in B and C includes two minor phases: as only one superconduct, then shielding fraction is not expected to be proportional to bright area.

The pseudo-monocrystalline character of K_*x*_Fe_2−*y*_Se_2_ (best visualized in the electron micrographs^21,29–31^ of Fig. 1) can be envisaged as a granular array wherein nano-sized superconducting granules of K_*s*_Fe_2_Se_2_ are randomly dispersed within the insulating K_2_Fe_4_Se_5_ matrix^17^. For such a granular configuration, one may apply the granular superconductor model^33^ so as to rationalize the evolution of the normal and superconducting properties of K_*x*_Fe_2−*y*_Se_2_. Within this simplifying scenario, we consider that a variation in control parameters (such as heat treatment, pressure *p*, and concentration *x*, *y*)^21,30,31,34^ modifies the size, distribution, separation, and concentration of metallic granules and that the latter modification can be expressed in terms of the model parameters. Accordingly, by probing the influence of control parameters on these parameters (such as the ones extracted from resistivity, as done here), one is able to construct a fundamental normal-state and superconducting phase diagram^33,35^, based on which it is possible to explain the control-parameter-induced manifestation of the followings: the high-*T* semiconducting-like character, the *T*_*mt*_ event, the *quadratic-in-T* Fermi-liquid like state, the multiple-*T*_*c*_ superconductivity, and how each of these states evolves (or being transformed into a neighboring state).

Below in Section II, we briefly discuss and summarize some theoretical expressions that are essential for describing the evolution of resistivity within the studied range of temperature, pressure and concentration. A detailed description of the granular model is given in the review of Beloborodov *et al*.^33^. In Section III, we apply these theoretical considerations so as to identify and understand the influence of control parameters on the granular character of K_*x*_Fe_2−*y*_Se_2_. Finally, we construct a generalized normal-state and superconducting phase diagram^33^ and discuss the evolution of its phase boundaries.

## A summary of basic resistivity expressions for a granular superconductor

Within the granular scenario^33^, the resistivity at specific thermodynamic condition [*ρ*(*T*, *x*, *y*, *p*), the main technique used in this work is a measure of the ability of electrons within a nano-sized granule to tunnel across the separating distance and Coulomb potential; quantitatively, *ρ*(*T*, *x*, *y*, *p*) is a function of the following model parameters: (i) the tunneling conductance *g* among metallic granules that are separated by insulating interface, (ii) the quantum confinement within each granule (with mean energy-level spacing *δ* and an inverse escape rate $\frac{h}{{k}_{B}{T}_{sat}}$ where ${T}_{sat}=\frac{g\delta }{{k}_{B}}$), (iii) Coulomb blocking potential *E*_*c*_ $({\rm{measured}}\,{\rm{by}}{T}_{cb}=\frac{g{E}_{c}}{{k}_{B}})$, (iv) the superconducting gap of single granule Δ, and (v) Josephson energy defined as *J* = *π*Δ*g*/2 which dictates whether Cooper pairs are delocalized $(J\gg {E}_{c})$ or localized $(J\ll {E}_{c})$.

The critical conductance *g*_*c*_ identifies the balance between the tunneling and the screened Coulomb blockade:

1 $${g}_{c}=\frac{1}{z\pi }\,\mathrm{ln}(\frac{{E}_{cb}}{\delta })=\frac{1}{z\pi }\,\mathrm{ln}(\frac{{T}_{cb}}{{T}_{sat}}),$$

*z* denotes the effective number of neighboring granules. A sample is an insulating if *g* < *g*_*c*_ while metallic if *g* > *g*_*c*_. For the particular *g* < *g*_*c*_ regime, there is an interesting situation wherein Cooper pairs tunneling (delocalization) overcomes the repulsion of *E*_*c*_ (localization). This defines another critical conductance

2 $${g}_{c}^{s}\approx {E}_{c}/{\rm{\Delta }}$$

such that coherent superconductivity occurs whenever $g > {g}_{c}^{s}$ even within the insulating ${g}_{c}^{s} < g < {g}_{c}$ regime.

Evidently, there are three different classes of *g*-regimes, namely: (i) *g* > *g*_*c*_, (ii) $g\ll {g}_{c}$ and (iii) ${g}_{c}^{s} < g < {g}_{c}$. In each, the evolution of normal and superconducting properties are distinct. In particular (see below), the thermal evolution of a *ρ*(*T*, *g*, *J*, *T*_*sat*_, *T*_*cb*_) curve within each *g*-regime is unique. On comparing the theoretical *ρ*(*T*, *X*) expressions with the experimental *ρ*(*T*, *x*, *y*, *p*) curves, one is able to determine the model parameters across the three regimes. This determination, together with the evolution with the control parameters, enables the construction of a generalized phase diagram across the wide range of experimental conditions.

### Granular metallic (*g* > *g*_*c*_) or homo*g*eneously disordered metallic ${\boldsymbol{(}}{\bf{g}}{\boldsymbol{\gg }}{{\bf{g}}}_{{\bf{c}}}{\boldsymbol{)}}$ regime

At higher conductance, $g\gg {g}_{c}$, screening reduces *E*_*c*_ and as a consequence charge tunnels easily leading to normal-state properties that are identical to those of a homogeneously disordered metal: Specifically, *ρ*(*T* → 0, *X*) → finite value and ∂*ρ*/∂*T* > 0. Similarly, if $J\gg {E}_{c}$, coherent bulk superconductivity will be established within *T* ≤ *T*_*c*_ (*E*_*c*_ → 0) → *T*_*c*,*bulk*_.

On the other hand for intermediate *g* > *g*_*c*_ regime, tunneling competes with Coulomb blockade leading to a characteristic nonmetallic *ρ*(*T*, *X*) with a thermal evolution which manifests four temperature regimes^33^: (i.1) When *k*_B_*T* is higher than Coulomb blockade, *ρ*(*T* > *T*_*cb*_, *g* > *g*_*c*_) is metallic (∂*ρ*/∂*T* > 0) though a disordered one. (i.2) Within the intermediate ${T}_{sat} < T < {T}_{cb}$ regime, resistivity manifests a characteristic *log-in-T* behavior:

3 $$\rho ({T}_{sat} < T < {T}_{cb},g > {g}_{c})=\frac{{\rho }_{cb}}{1-\,\frac{1}{z\pi g}\,\mathrm{ln}(\frac{{T}_{cb}}{T})}$$

where *ρ*_*cb*_ = *ρ*(*T* = *T*_*cb*_). (i.3) Within the low temperature range *δ*/*k*_B_ < *T* < *T*_*sat*_, resistivity is due to two contributions: a saturated term [based on Eq. (3)] and Altshuler-Aronov-type contribution:

4 $$\rho ({T}_{c}^{onset} < T < {T}_{sat},g > {g}_{c})=\frac{{\rho }_{cb}}{1-\frac{1}{z\pi g}\,\mathrm{ln}(\frac{{T}_{CB}}{{T}_{sat}})+\frac{1.83}{2z{\pi }^{2}g}\sqrt[2]{\frac{T}{{T}_{sat}}}}{\rm{.}}$$

(i.4) The granular superconducting regime: As that *g* > *g*_*c*_ > ${g}_{c}^{s}$ and *J* > *E*_*c*_ due to screening, superconductivity always emerges in this granular regime. The onset temperature ${T}_{c}^{onset}$ is higher than the zero-resistivity point ${T}_{c}^{zero}$^ 1^: Within the so-called superconducting fluctuation region, ${T}_{c}^{zero} < T < {T}_{c}^{onset}$, no global coherence is established.

Experimental studies within the intermediate *g* regime of *A*_*x*_Fe_2−*y*_Se_2_ compounds, (see below) revealed (i.a) a surge of a *log-in-T* character, (i.b) an absence of a thermal evolution similar to Eq. 4 (onset of coherence well above the saturation regime: *T*_*mt*_ > *T*_*sat*_), (i.c) a *quadratic-in-T* character, and (i.d) a granular superconductivity.

### *g* < *g*_*c*_ granular insulator regime

Here, metallic granules are widely separated, tunneling conductance is weak and as such Coulomb blockade is weakly screened; this leads to the insulating *ρ*(*T* → 0) → ∞ character which is completely different from the conventional band-gapped insulating case. It is also different from the Mott-type variable range hopping case: The involved Coulomb charging potential, *E*_*c*_, works against tunneling to neighboring grains.

It is recalled that, as g → g_c_, an increase in *g* tends to promote the screening of *E*_*c*_ such that at *g*_*c*_ (Eq. 1) a metal-insulator transition takes place. Within this insulating range, *g* < *g*_*c*_, one identifies two regimes namely (ii.1) $g\ll {g}_{c}$ and (ii.2) ${g}_{c}^{s} < g < {g}_{c}$.

### ${\bf{g}}{\boldsymbol{\ll }}{{\bf{g}}}_{{\bf{c}}}$ regime

For temperatures higher than a characteristic temperature *T*_*AV*_, the thermal hopping among only nearest neighbors is effective leading to an Arrhenius-type resistivity:

5 $$\rho (T > {T}_{AV},g\ll {g}_{c})={\rho }_{{\rm{A}}}exp({T}_{{\rm{A}}}/T\mathrm{).}$$

On the other hand, when thermal energy is lowered to below *k*_*B*_*T*_*AV*_, charge transport is dominated by electronic tunneling to far-apart granules that have energies close to Fermi level; in close similarity to Efros-Shklovskii process in amorphous semiconductors, this leads to

6 $$\rho (T < {T}_{AV},g\ll {g}_{c})={\rho }_{{\rm{E}}{\rm{S}}}{\exp }(\sqrt[2]{{T}_{{\rm{E}}{\rm{S}}}/T})$$

wherein *T*_ES_ depends on the characteristic granular conditions. Within this $g\ll {g}_{c}^{s} < {g}_{c}$ regime, no superconductivity will be manifested.

#### ${g}_{c}^{s} < g < {g}_{c}$ regime: the superconducting insulator transition

$\rho (T\gg {T}_{c},{g}_{c}^{s} < g < {g}_{c})$ follows the same thermally-activated evolution as that shown in Eqs 5 and 6. As far as the superconductivity is concerned, Eq. 2 indicates that if *E*_*c*_ is reduced by screening (recall that *g* ≠ 0) to the extent that *J* > *E*_*c*_, then on further cooling, a superconducting state would emerge: This marks the exotic superconducting-insulator transition^35^. Such a transition is wide and incomplete; more often $\rho (T < {T}_{c}^{onset},{g}_{c}^{s} < g < {g}_{c}){\nrightarrow}0$.

It is worth mentioning that not all K_*x*_Fe_2−*y*_Se_2_ samples show this normal-state insulating-like behavior; if manifested, it is possible to transform it into a granular metallic-like behavior by a suitable manipulation of a control parameter so as to increase *g* or reduce *E*_*c*_. Similarly, for the granular superconductivity, such manipulation would lead to sharpening of the transition width and enhancement of *T*_*c*_: i.e. transformation from phase-fluctuating intra-grain superconductivity into a globally coherent, bulk, superconductivity.

### Procedures for analysis of a resistivity of granular K_x_Fe_2−y_Se_2_

Based on the above theoretical arguments, one classifies a sample as a granular metal [*g* < *g*_*c*_, Eq. (3)] if its *ρ*(*T* → 0, *control*) → finite while as a granular insulator (*g* < *g*_*c*_, Eqs 5 and 6) if its *ρ*(*T* → 0, *control*) → ∞ (*conrol* = heat treatment, *x*, *y*, or *p*). Influence of each control parameter can be followed by monitoring the corresponding variation in *ρ*(*T*, *control*) curves. On fitting experimental *ρ*(*T*, *control*) to one of the above theoretical *ρ*(*T*, *X*) expressions, one obtains the involved parameters and as such their evolution: *X* (*control*). On the other hand, one identifies the following events from the thermal evolution of *ρ*(*T*, *control*): ${T}_{c}^{onset}$, ${T}_{c}^{zero}$, *T*_mt_ (the maximum of the hump, the point below which metallicity emerges), *T*_*cb*_ (see Eq. 3), *T*_*AV*_ (crossover point from Arrhenius-type resistivity, Eq. 5, into VRH-type resistivity, Eq. 6), *T*_*int*_ (above which the semiconducting feature of the matrix is dominant). The following events can be estimated from literature^21,29,30,36,37^: *T*_*N*_ (Néel point of magnetic transition), *T*_*ps*_ (the point of phase segregation) and *T*_*vo*_ (Fe-vacancy order point). Below, all points are plotted against the conductance *g* which is obtained from a fit of Eq. (3) to measured *ρ*(*T*, *control*) curves: each obtained *g* − *T* phase diagram is discussed as being a projection of the generalized *g* − *E*_*c*_/Δ − *T* diagram^33,35^, the latter is most appropriate for the description of the normal and superconducting properties.

## Results

### Influence of quenching on granularity of K_0.8_Fe_2_Se_2_

Granularity of K_*x*_Fe_2−*y*_Se_2_ develops, below the segregation point *T*_*ps*_, as a remnant of the high-*T*, *I4/mmm* phase^21,29,30,36,38^. This work followed and evaluated the influence of heat-treatment on this granular character by subjecting four identically synthesized K_0.8_Fe_2−*y*_Se_2_ samples^38–40^ to slightly different quenching stages (see Materials and measuring techniques). Below we show that such a slight variation in quenching procedures does bring about strong modification in their microstructural, elemental, structural, resistive and magnetic properties.

#### Microstructure, composition and crystal structure

Figure 1 shows the microstructures of freshly cleaved surfaces of the four samples. In agreements with refs^21,30,36,41^. all images exhibit metallic (insulating) regions as a mesh-like bright-textured (dark) area. The average size (possibly also Fe-content) of each individual stripe-like metallic granule determines its average individual-granules-related properties (e.g. ${T}_{c}^{onset}$) while their spatial separation and distribution determine *g*, *E*_*c*_ and the overall bulk properties (e.g. ${T}_{c}^{zero}$ and superconducting shielding fraction). Table 1 indicates that the average Fe-content within the superconducting granules decreases progressively along the sequence of D, A, B to C^40^: Evolution of ${T}_{c}^{onset}$ follows a similar monotonic decrease.

**Table 1:** Representative numerical parameters of studied A, B, C and D samples as obtained from diffraction analysis (*c*-axis parameters), compositional analysis (ratio of the estimated superconducting phase), Kondo-type process (*T*_*K*_ and *F*) and resistivity fit parameter at 400 K (*ρ*_*cb*_ in Eq. 3).

	Quench A	Quench B	Quench C	Slow-cool D
*c-*parameter of major phase (Å)	14.138	14.109	14.110	14.133
*c-*parameter of minor phase (Å)	14.246	14.222	14.204	14.261
	—	14.375	14.379	—
compositional ratios of SC phase	K_0.35_Fe_1.83_Se_2_	K_0.53_Fe_1.74_Se_2_	K_0.58_Fe_1.71_Se_2_	K_0.40_Fe_1.95_2Se_2_
localization *T*_*K*_(K)	—	50.6	52.3	—
localization *F* (*m*Ω-cm) [Eq. (7)]	—	85	223	—
${\rho }_{cb}$ (*m*Ω-cm) [Eq. (7)]	0.08154	0.15151	0.20877	1.41976

**Figure 2:**
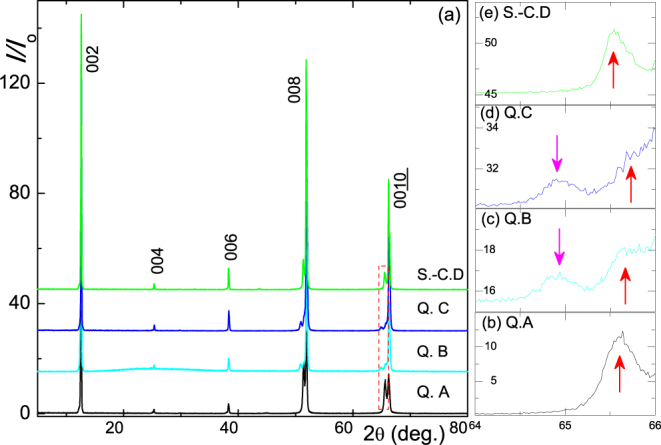
(**a**) X-ray diffractograms of the four pseudo-monocrystals demonstrating the (00*l*) Bragg peaks of the major and minor phases^16,21^ in each heat-treated K_0.8_Fe_2_Se_2_. The calculated *c*-*axis* parameters are given in Table 1. Panels (b)–(e): expansions of the minor-phase (0010) peaks for samples Q.A (quench A), Q.B (quench B), Q.C (quench C) and S.-C.D (slow-cool D), respectively (see text); here, the upward arrows emphasize the shorter *c-parameter* peak while the downwards emphasize the longer *c-parameter* peak.

**Figure 3:**
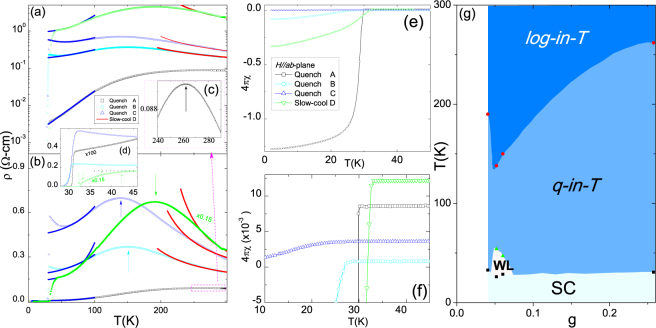
(**a**,**b**) ρ(T, Z) curves (*Z* = A, B, C, D). The solid high-*T*, red (low-*T*, blue) lines are fits to Eqs (3 and 7). Short vertical arrows mark the coherence event at *T*_*mt*_. (**c**) Expansion of the high-*T* regime of *ρ* (*T*, *A*) curve around *T*_*mt*_. (**d**) Low-*T* expansion of all curves around *T*_*c*_. Vertical arrows indicate three onset points ${T}_{c}^{onset1}$ ≈ 42 K, ${T}_{c}^{onset2}$ ≈ 37 K, ${T}_{c}^{onset3}$ ≈ 33 K and one ${T}_{c}^{zero}$ ~32 K. (**e**) Thermal evolution of normalized ZFC susceptibility of the four pseudo-monocrystals. (**f**) Low-*T* expansion of all curves around ${T}_{c}^{mag}$ (onset of diamagnetism)^21,30,31,34^. Values of 4 *πχ* should be considered as indicative since both the density and molecular weight are not precisely determined. (**g**) *g* − *T* phase diagram of the studied samples. *log-in-T*: the granular behavior governed by Eq. (3). *quadratic-in-T*: Koshino-Taylor contribution (third term of Eq. 7 and blue solid line in panel b). SC denotes superconducting (${T}_{c}^{zero}$) phase while WL the above-mentioned Kondo-like or weak localization regime [last term of Eq. (7)].
(iv) *The superconducting regime*: Fig. 3(b,d) indicate that all four samples superconduct^30,31,36^; within the context of the granular model, this implies that $\delta \ll {\rm{\Delta }}$ (Anderson criterion) and that $J\gg {E}_{c}$ and of course $g > {g}_{c}^{s}$. In addition, Fig. 3(d) reveals multiple SC transition points^30,31,36^; as an example, sample D exhibits ${T}_{c}^{onset1}$ ≈ 42 K refs^4,5,29,31^, ${T}_{c}^{onset2}$ ≈ 37 K and ${T}_{c}^{onset3}$ ≈ 33 K; this is attributed to a nonuniform distribution of Fe concentration within the superconducting granules^3^. On average, ${T}_{c}^{onset}$ of a slow-cooled sample is higher than that of a quenched one and that ${T}_{c}^{zero}$ decreases along D, A, B and C; for quenched samples, this follows the evolution of *g*, *J* and Fe-content.

**Figure 4:**
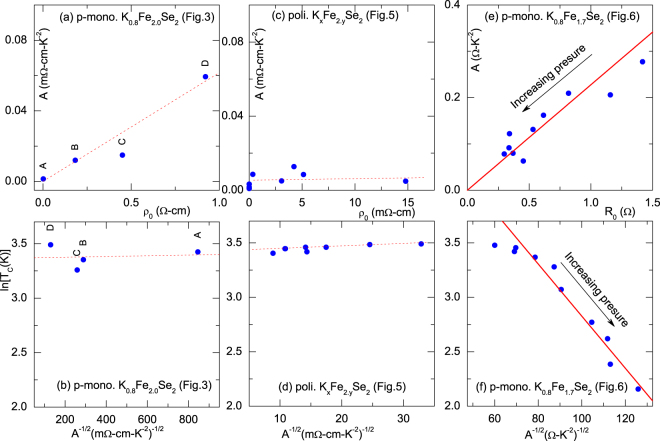
(**a**,**c**,**e**) Plots  of *A* versus *R*_*o*_ (or *ρ*_*o*_) as obtained from fits of Eq. 7. In panels (**a**,**c**,**e**), the error bars were found to be less than the size of the symbols: this observation is valid for other fit parameters of Eq. 7. Solid line in panel (e) is a linear fit to Eq. 8 wherein *B* = 2.28 × 10^−4^ K^−2^. (**b**,**d**,**f**) Plots of *ln*(*T*_*c*_) *versus* [*A*_*tot*_]^−1/2^. Away from the unreliable low-pressure range, the solid line in panel (f) is a fit to a linearized Eq. 9 with Θ = 185 ± 15 K and ${\mathcal F}$ = 42 ± 4 Ω^1/2^ K^−1^. Sample type as well as figure number (from which the parameters were taken) are shown in each panel.

For samples Quench B and C, two *c*-*axis* parameters are shown, reflecting the two minor peaks of Fig. 2(c,d). All other parameters (e.g. ${T}_{c}^{zero}$ and ${T}_{c}^{mag}$, *A* and *ρ*_*o*_) are shown either in Figs 3 and 4.

A careful look at Fig. 1 indicates that granules of slow-cool sample D are considerably large in size but are well separated^21,30,36,41^: Since the probability amplitude of tunneling decays exponentially with distance and since the average separating distance of sample D is much larger than that of the quenched samples, then its *g* must be the smallest. On the other hand, the average separation distance of bright granules of quenched samples are monotonically increased along the sequence A, B to C. It is, then, expected that *g* is the highest for A and decreases monotonically along A, B, C, D.

The single-crystal diffractograms (see Fig. 2 and Table 1) consist of the (00*l*) Bragg peaks of both the major and minor phases^16,22^. A closer look at intensities of the minor-phase, Fig. 2(b–e), reveals that the (00*l*) peak of Quench A and Slow-cool D is single and relatively sharp. In contrast, for Quench B and C, there are two broad, relatively small, and closely-situated peaks: the shorter *c-parameter* peak, upward short arrow in Fig. 2(b–e), is evident in all samples and can be safely related to the Fe-rich superconducting K_*s*_Fe_2_Se_2_ phase. The longer *c-parameter* peak (downward arrow) is related to the so-called third phase that was identified by Ricci *et al*.^17^; its presence, in contrast to the second phase, is manifested prominently only in the relatively fast-quenched samples.

#### Electric resistivity

Figure 3(a) shows the thermal evolution of in-plane *ρ*(*T*, *Z*) (*Z* = A, B, C, D) which increases monotonically along A, B, C and D; this is consistent with the decreasing *g* deduced from Fig. 1 (also reminiscent of the heat-treatment reported in refs^29,30^. For all samples, *g* < *g*_*c*_ and, moreover, the overall thermal evolution of *ρ*(*T*, *Z*) is similar in that it traverses, successively, a *log-in-T* → *peak centered  at T*_mt_ → *quadratic-in-T* → *superconducting* state:

(i) *The log-in-T regime* (∂*ρ*/∂*T* < 0, *T*_mt_ < *T* < 300 K) is related to the granular character; indeed all *ρ*(*T* < *T*_*mt*_, *Z*) curves follow satisfactorily Eq. 3.
(ii) *The T*_mt_-*peak regime* is evident in all samples, in particular the metal-like sample A [see Fig. 3(b)]. Two earlier interpretations of this *T*_mt_ event were suggested: one is related to an onset of an orbital selective Mott transition^11^ while the other is related to an onset of percolated K_*s*_Fe_2_Se_2_ conducting filament within an insulating K_2_Fe_4_Se_5_ background^16,17^. Considering the granularity revealed in Fig. 1, the successful *log-in-T* fits of Fig. 3 and the observed strong correlation of *T*_mt_ with *g* [Fig. 3(g)], we attribute this *T*_mt_ event to an onset of coherence or to a gradual increase in *g* such that below *T*_mt_ it becomes higher than *g*_*c*_. The net effect is a crossover into a homogeneously disordered metallic behavior (percolative conductivity being a limiting case). A possible increase in *g* may arise from an increase in the average size of the metallic granules; such an increase was reported by Ricci *et al*.^17^.
(iii) *The quadratic-in-T regime*^10^ is exhibited over the wide ${T}_{c}^{onset} < T < {T}_{mt}$ range in both A and D but is masked in each of B and C by a competing low-*T* Kondo-like process (${\rho }_{{\rm{K}}}=Fln(\frac{{T}_{{\rm{K}}}}{T}$). It is recalled that the granular configuration is still maintained below *T*_mt_, only that the *log-in-T* state of Eq. 3 is transformed below *T*_*mt*_ into a homogeneously disordered metal-like state. We attribute this *quadratic-in-T* behavior to an inelastic scattering from defects^42^ such as granules boundaries or any residual impurity: A Koshino-Taylor contribution^42^ having *ρ*_KT_ = *AT*^2^. Then, the total resistivity is
  7 $$\rho (T)={\rho }_{{\rm{res}}}+{\rho }_{{\rm{ph}}}+{\rho }_{{\rm{KT}}}+{\rho }_{{\rm{K}}}={\rho }_{o}+\beta {T}^{5}+A{T}^{2}+F\,\mathrm{ln}\,(\frac{{T}_{{\rm{K}}}}{T}),$$
  where in *ρ*_ph_ = *βT*^5^ approximate the phonon contribution and, from above, the coefficient *A* and the residual resistivity *ρ*_*o*_ are linearly related ^42^:
  8 $$A=B{\rho }_{o}.$$
  The parameters of the *quadratic-in-T* fit of Fig. 3(a) are shown in Fig. 4(a,b) while those of Kondo-like fits are shown in Table 1.

Based on the analysis of the thermal events and of the corresponding *g* [Fig. 3(b)], we constructed the *g* − *T* phase diagram of Fig. 3(g).

#### Magnetic susceptibility

Normalized zero-field cooled (ZFC) susceptibilities (${H}_{\parallel ab}$ = 10 Oe) of all samples are shown in Fig. 3(e,f). Our main interest here is focused on quench-dependent evolution of ${T}_{c}^{mag}$ and superconducting shielding fraction, ${V}_{sc}^{mag}$. ${T}_{c}^{mag}$, Fig. 3(f), is in satisfactorily accord with ${T}_{c}^{zero}$ and both, as mentioned earlier, are correlated with *g* [obtained from Fig. 3(b)] and Fe-content (Table 1). The ${V}_{sc}^{mag}$ fraction, on the other hand, decreases dramatically along *A* → *D* → *B* → *C*. Considering the quenched samples, this sequence is consistent with the evolution of *g* and Fe-content: accordingly, the third phase in Fig. 2 is identified as a normal conductor and its presence is considered to be nonessential for (if not detrimental to) superconductivity. The relatively high ${V}_{sc}^{mag}$, ${T}_{c}^{zero}$ and ${T}_{c}^{mag}$ of the slow-cooled sample D is most probably related to an increase in *J* or Fe-content which may compensate for its lower *g*.

### Influence of concentration variation on granularity of K_*x*_Fe_2−*y*_Se_2_

**Figure 5:**
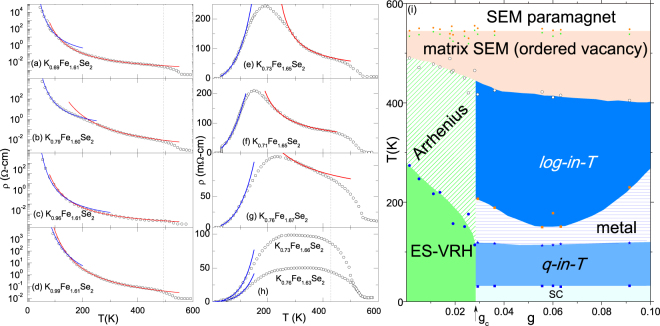
(**a**) Representative analyzed *ρ*(*x*, *T*) curves of polycrystalline K_*x*_Fe_2−*y*_Se_2_ (resistivity curves were taken from Yan *et al*.^43^). (**a**–**d**) Analyzed $\rho (T,g\ll {g}_{c})$ curves of the granular insulator regime. The solid red (blue) lines are fits to Eqs (5 and (6)). The effective *g* for granular insulators was calculated^33^ via $g=\frac{1}{\pi z}\,{ln}(\frac{{E}_{c}}{{{\rm{\Delta }}}_{m}})$ wherein *E*_*c*_ ≈ 2100 K and Δ_*m*_ is Mott-like activation energy obtained from Arrhenius fits. (**e**–**g**) Representative *ρ*(*T*, *g* < *g*_*c*_) curves within the granular metal regime. The solid red (blue) is a fit to Eqs (3 and (7)). (**h**) Representative curves of $g\gg {g}_{c}$ regime: Effective *g* was calculated^33^ using $g=\frac{G}{a.{\rho }_{400{\rm{K}}}}$ where *G*, *a*, and *ρ*_400K_ are, respectively, conductance quantum, average diameter of metallic granules (100 Å), and resistivity at 400 K. Vertical thin dashed lines, in panels (**a**–**h**), separate matrix semiconductivity from *log-in-T* character. (**i**) *g* − *T* phase diagram of polycrystalline K_*x*_Fe_2−*y*_Se_2_. *Arrhenius*: an Arrhenius process [Eq. (5)]; ES-VRH: ES-type variable range hopping process [Eq. (6)]. *log-in-T*: the granular behavior governed by Eq. (3). *quadratic-in-T*: Koshino-Taylor contribution of Eq. 7. SC: the superconducting phase. As can be seen in panels (**a**–**h**), there are two high-temperature regimes: one is related to semiconducting matrix within 400 K < *T* < ${T}_{ps}\simeq 520\,{\rm{K}}$ and the other is the $T > {T}_{vo}\simeq 580\,{\rm{K}}$ paramagnetic regime.

To verify the generality of our analysis, let us apply the granular model for the analysis of *ρ* (*T*, *x*, *y*) of the polycrystalline K_*x*_Fe_2−*y*_Se_2_ samples reported in Yan *et al*.^43^. The analyzed *ρ*(*T*, *x*, *y*) curves, Fig. 5, fall into two different *g* classes (see above):

(i) The granular insulating $g\ll {g}_{c}$ regime, represented by Fig. 5(a–d). Indeed, no superconductivity is evident. Furthermore, *ρ*(*T*, *x*, *y*) curves were analyzed according to Eqs (5 and 6) namely an ES-type hopping regime below *T*_*VA*_ ≈ 100–300 K and an activated regime within *T*_*VA*_ < *T* < 400 K. For 400 K < *T* < *T*_*N*_^21,29,30,36^, the intrinsic semiconductivity of the K_2_Fe_4_Se_5_ matrix dominates^16,44^. Here, any defects, e.g. excess Fe, within the semiconducting K_2_Fe_4_Se_5_ matrix may act as carrier dopant^22^.
(ii) The granular metallic *g* > *g*_*c*_ regime which is represented by Fig. 5(e–g). Here, one identifies the above-mentioned four *T* regimes: *log-in-T* behavior [Eq. (3)], *T*_mt_-peak*, quadratic-in-T*  [Eq. (7)] character and superconductivity. It is worth mentioning that the curves of Fig. 5(h) exhibit a $g\gg {g}_{c}$ homogeneously disordered behavior.

The obtained model parameters together with all thermal events (including *T*_*N*_, *T*_*ps*_ and *T*_*vo*_) were used to construct the *g* − *T* phase diagram of Fig. 5(i). Furthermore, the fit parameters of *quadratic-in-T* fit of Fig. 5(e–h) are shown in Fig. 4(c,d). No Kondo-like contribution is detected: an indication that it is an extrinsic effect.

The evolution of *ρ*(*T*, *x*, *y*) curves in Fig. 5(a–h) is controlled by *x* and *y* which in turn control *g*; the evolution of *g* is accompanied by a transformation of the insulating *g* < *g*_*c*_ behavior into the metallic (*g* > *g*_*c*_ or $g\gg {g}_{c}$) character. The data reported by Yan *et al*.^43^ did not show any curve belonging to the intermediate ${g}_{c}^{s} < g < {g}_{c}$ range. Nevertheless, curves belonging to this range were reported for isomorphous (K, Tl)_*x*_Fe_2−*y*_Se_2_ by Fang *et al*.^3^: indeed their Fig. 3(a) shows a superconducting-insulator-transition occurring within the Fe concentration range of 1.68 < 2 − *y* <1.69.

### Influence of pressure on granularity of K_0.8_Fe_1.7_Se_2_

Just as in the preceding sections, we applied the granular model to the analysis of *ρ*(*T*, *p* < 9 GPa) curves of pseudo-monocrystalline K_0.8_Fe_1.7_Se_2_ as reported by Guo *et al*.^10^. Evidently the ambient-pressure curve exhibits, unambiguously, the above-mentioned four thermal regimes with no vestige of granular insulating character or Kondo-like behavior: for *p* < 9 GPa, we obtained *g* > *g*_*c*_ and *J* > *E*_*c*_.

**Figure 6:**
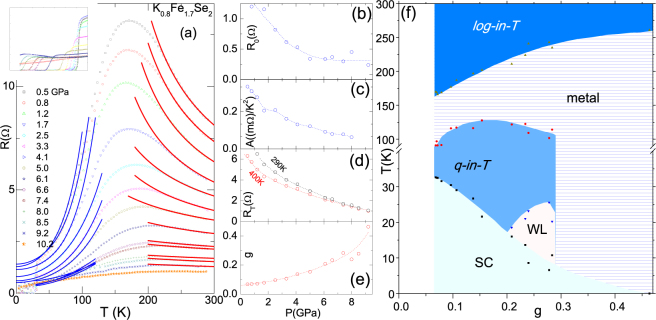
(**a**) Representative isobaric *ρ*(*T*, *p*) curves of pseudo-monocrystalline K_0.8_Fe_1.7_Se_2_ (resistivity curves were taken from Guo *et al*.^10^). Solid red (blue) is a fit to Eqs (3 and 7). *ρ*(T, 9.2 GPa) is a representative of the disordered metallic state. *Inset*: Expanded low-temperature curves^10^ showing the localization behavior. (**b**) *R*_*o*_ *versus p* [Eq. (7)]. (**c**) A *verus p* [Eq. (7)]. (**d**) Fit parameter *R*_400K_ and exoerimental *R*_300K_ *versus p*. (**e**) *g versus p* [Eq. (3)]. (**f**) *g* − *T* phase diagram as obtained from analysis of thermal transition/crossover events appearing in panel (a)^10^. Interestingly, no activated contributions are evident implying that *g* > *g*_*c*_ for all curves. The *log-in-T*, *quadratic-in-T*, metal, and SC have their usual meaning. WL denotes Kondo-like or weak localization as manifested in inset of panel (a).

The fit curves of the granular character (Eq. 3) as well as those of Koshino-Taylor contribution (Eq. 7) are shown in Fig. 6(a). The baric evolution of these parameters are shown in Fig. 6(b–e); evidently, as pressure is increased up to 9 GPa, *g*(*p*) is systematically enhanced and, concomitantly, all *R*_o_(*p*), *A*(*p*) and *R*_*T*_(*p*) are monotonically decreased. Furthermore, *T*_*mt*_ is increased while *T*_*c*_ is decreased. All thermal events are plotted on the *g* − *T* phase diagram of Fig. 6(f).

We did not extend the granular superconducting scenario to the *p* > 9 GPa regime because, for above 9 GPa, (i) the structural symmetry is transformed from *I4/m* into *I4/mmm* (signaling two distinct *g* regimes), (ii) the resistivity is strongly flattened at high-temperature (conversion of a *log-in-T* into a homogeneously disordered metallic contribution), (iii) a Kondo-like behavior is manifested at lower temperature (masking the *quadratic-in-T* contribution), and (iv) although the low-pressure superconductivity is being monotonically suppressed, a re-entrant superconducting state emerges above 9 GPa (this can not be straightforwardly related to the baric evolution of *g*).

## Discussion and Summary

**Figure 7:**
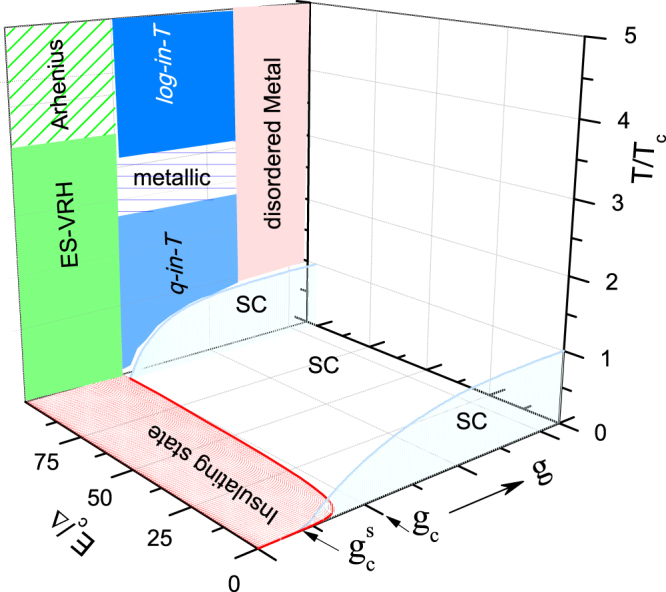
A sketch of a generalized *g* − *E*_*c*_/Δ − *T* phase diagram of a granular superconductor *A*_*x*_Fe_2−*y*_Se_2_ [adapted from Figs 4 and 17 of ref.^33^. Copyright (2007) by the American Physical Society]. None of the axes was drawn to scale. Within the normal state, *ES-VRH*: ES-like variable range hopping, Eq. (6); *Arrhenius*: activated resistivity, Eq. (5); *log-in-T*: the log-in-T resistivity of Eq. (3); *q-in-T:* Koshino-Taylor *quadratic-in-T* contribution, Eq. (7); *metallic, disordered metal*: metallic regime wherein *g* > *g*_*c*_, $g\gg {g}_{c}$. Two *T*_*c*_ (*g*) curves are shown: Back (front) curve illustrates ${E}_{c}/{\rm{\Delta }}\gg$ 1 (${E}_{c}/{\rm{\Delta }}\ll 1$). ${g}_{c}^{s}$, Eq. (2), marks the boundary separating the superconducting from the insulating phases. Within the zero-temperature projection, solid red line separates the superconducting from the insulating states^35^: A sample located at the right of this line superconducts.

The similarity of the *g* − *T* phase diagrams of Figs 3(g),5(i) and 6(f) can be taken as a confirmation of the adequacy and elegance of the analysis in terms of a granular superconductor model. A generalization of these phase diagrams is shown in Fig. 7 which demonstrates, in addition to the *g* − *T* projection, the evolution along the third axis $\frac{{E}_{c}}{{\rm{\Delta }}}$.

With no loss of generality the above analysis was carried out assuming *E*_*c*_ and Δ to be constant. In spite of such a simplification, one observes that a variation in *g* leads to successive transformations, among various electronic states; such transformations are manifested in polycrystals and in pseudo-monocrystals when any of the different control parameters is varied. This highlights the merit and success of the adopted model: it rationalizes, in terms of few fundamental parameters, the normal and superconducting properties of various Fe-based chalcogenides (see e.g., refs^1–8,16,38,39^. Such a generalization was demonstrated earlier for BiS_2_-based superconductors^45^.

A closer comparative look at Figs 3(g),5(i),6(f) and 7 reveals that, in spite of the straightforward rationalization of the normal-state phase boundaries of all samples in terms of the granular metal model, the evolution of *T*_*c*_(*g*) needs a further clarification: While Figs 3(g) and 5(i) can be situated within the *g* − *T* region wherein *T*_*c*_ is weakly modified, *T*_*c*_ (*g*) of Fig. 6(f) manifests a strong reduction which must be driven by another competing mechanism that overrides the evolution predicted by granular superconductor scenario. Dome-like evolution may be obtained if it is possible to include a negative pressure range wherein the contributions of these competing mechanisms are inverted.

There is another more subtle difference among the various phase diagrams: how each control parameter modifies the correlation between the superconductivity (as reflected in *T*_*c*_) and the *quadratic-in-T* contribution (as reflected in *A*)? and how both *T*_*c*_ and *A* are influenced by disorder/defects (as measured by *R*_*o*_)? Let us consider first the the pressure-dependence: Fig. 4(e) demonstrates that *A*(*p*) is linearly correlated with *R*_*o*_(*p*) confirming the Koshino-Taylor relation given in Eq. (8). On the other hand, Fig. 4(f) reveals that, within the reliable 1 < *p* < 9 GPa range, *T*_*c*_ is correlated to *A* by^46–48^

9 $${T}_{c}={\rm{\Theta }}\,\exp \,(-\frac{1}{ {\mathcal F} \sqrt{A}}),$$

where Θ and ${\mathcal F}$ are sample-dependent constants. This relation suggests a common scenario for both superconductivity and *quadratic-in-T* contribution^48–50^ and, furthermore, this common scenario must involve the Koshino-Taylor process. It is worth adding that [in stark contrast to the strong baric dependence of *T*_*c*_(*p*), *R*_T_(*p*) and *A*(*p*), shown in Fig. 4(e and f)] there is no similar correlation between *T*_*c*_ and *A* or between *A* and *R*_0_ within either the quench [Fig. 4(a and b)] or the concentration variation [Fig. 4(c and d)]. This is most probably related to how each control parameter influences the scattering processes. Evidently, a Koshino-Taylor contribution, being an inelastic process, is most strongly modified by pressure [which also modifies *T*_*c*_ and *R*_0_ (*p*)]. In contrast, a variation in quenching or in concentration does introduce additional elastic scattering processes which (probably, being an Anderson-type) hardly modifies *T*_*c*_.

In summary, we modeled K_*x*_Fe_2−*y*_Se_2_ system as a granular superconductor wherein nano-sized superconducting K_*s*_Fe_2_Se_2_ granules are embedded within the insulating continuum of K_2_Fe_4_Se_5_. Based on this scenario, the influence of heat treatment, concentration and pressure is considered as a manipulation of size, distribution, separation and Fe-content of the metallic granules. These can be followed in terms of the model parameters such as tunneling conductance, the Coulomb charging energy, and Josephson energy. We showed that this model explains satisfactorily the evolution of normal-state and superconducting phase diagram of polycrystalline as well as pseudo-monocrystalline K_*x*_Fe_2−*y*_Se_2_ (and by extension *A*_*x*_Fe_2−*y*_Se_2_) systems when any of the various control parameters is modified. A generalized phase diagram is constructed.

### Materials and Measuring Techniques

Single crystals with a nominal composition K_0.8_Fe_2.0_Se_2.0_ were grown by the one-step method^29,39^ (more details were given in ref.^40^). For studying the influence of quenching procedures on the microstructural, elemental, structural, resistive and magnetic properties, we identically synthesized four samples^40^ and afterwards subjected them to slightly different quenching stages, starting from 700 °C: (i) Quench A: a single crystal, sealed in evacuated quartz tube, was quenched directly into water. (ii) Quench B: similar to Quench A but quenched into iced water. (iii) Quench C: a single crystal (using a carbon crucible and sealed Ar-filled stainless steel tube) was quenched into iced water. Finally, (iv) Slow-cool D: a single crystal, evacuated and sealed, was slowly-cooled.

Room-temperature, Cu *K*_*α*_ X-ray minifelx-type diffractometer was used for structural characterization. The microstructures were obtained by back-scattered electron images of a scanning electron microscope (SEM; JSM-6010, JEOL) operated at 15 kV. The compositional ratio was analyzed by energy dispersive X-ray (EDX) spectroscopy attached to the same SEM equipment. EDX area analysis (together with the microstructural BSE image analysis) were used for calculating the K:Fe compositional ratio within an averaged area of dark and bright domains^40^. The estimated compositional ratios of the superconducting phase are given in Table 1. DC in-plane electrical resistivities were measured by a standard four-probe method while magnetization by a superconducting quantum interference device (SQUID) magnetometer. At every stage, sample manipulations were handled exclusively in a glovebox operated under Ar atmosphere.
